# Coexistence of Fahr‐Type Intracranial Calcification and a Presumed Cerebellar Pilocytic Astrocytoma With Obstructive Hydrocephalus: A Rare Case Report

**DOI:** 10.1002/ccr3.73552

**Published:** 2026-09-16

**Authors:** Bikramaditya Prasad Sah, Kashif Qureshi, Murtaja Satea, Khaled Fares AlAli, Maren Ruka, Muhammad Mohsin Khan, Tural Rajabov, Bipin Chaurasia

**Affiliations:** ^1^ Neurosurgery Department Madan Bhandari Academy of Health Sciences, Hetauda Hospital Hetauda Nepal; ^2^ Department of Neurosurgery Yale School of Medicine New Haven Connecticut USA; ^3^ Department of Neurosurgery, College of Medicine University of Warith Al‐Anbiyaa Karbala Iraq; ^4^ Department of Neurosurgery Zayed Military Hospital Abu Dhabi UAE; ^5^ Department of Neurosurgery Mother Theresa Hospital Tirana Albania; ^6^ Department of Neurosurgery Hamad Medical Corporation Doha Qatar; ^7^ Liv Bona Dea Hospital Baku Azerbaijan; ^8^ Department of Neurosurgery Neurosurgery Clinic Birgunj Nepal

**Keywords:** astrocytoma, Fahr disease, hydrocephalus, posterior fossa

## Abstract

A 18‐year‐old female presented with recurrent vomiting and headaches. Neuroimaging revealed extensive bilateral intracranial (Fahr‐type) calcification together with a cerebellar mass lesion. She underwent excision of the cerebellar mass. Etiology of the calcification could not be established, and histopathology showed only hyalinized acellular tissue, leaving the diagnosis of pilocytic astrocytoma presumptive.

## Key Clinical Message

The coexistence of Fahr calcification and a presumed cerebellar pilocytic astrocytoma is rare. Because neither the etiology of the calcification nor the tumor diagnosis could be confirmed, this case highlights the importance of considering more than one pathology when symptoms are progressive and disproportionate to a single diagnosis.

## 1 Introduction

Fahr's disease, also known as primary familial brain calcification (PFBC), is a rare neurological disorder affecting young to middle‐aged adults. The two designations are not interchangeable: Fahr disease, or PFBC, denotes primary calcification with no identifiable secondary cause, whereas Fahr syndrome refers to calcification arising secondary to an underlying disorder, most commonly hypoparathyroidism or pseudohypoparathyroidism [1, 2]. The exact cause of this condition is not known but commonly found to be associated with a variety of conditions, e.g., endocrine disorders, mitochondrial myopathies, dermatological abnormalities and infectious diseases. Coexistence of Fahr's disease with brain tumor is associated with an even lower incidence. Abnormal deposition of calcium carbonate and calcium phosphate occurs commonly in the basal ganglia, thalamus, hippocampus, cerebral cortex, cerebellar subcortical white matter, and dentate nucleus [1, 3, 4, 5, 6].

These intracranial calcifications are typically associated with movement disorders, psychiatric symptoms, cognitive impairment, and seizures. Pathogenesis is most commonly linked to mutations involving calcium metabolism and disturbances in the neurovascular unit, leading to progressive mineral deposition within the brain [1, 7, 8, 9]. Pathogenic variants in SLC20A2, PDGFB, PDGFRB, XPR1, MYORG, and JAM2 have been implicated, several of which disrupt inorganic phosphate homeostasis and the integrity of the neurovascular unit at the blood–brain barrier [5].

Pilocytic astrocytoma, in contrast, is a World Health Organization (WHO) Grade I glioma that primarily affects children and young adults. It is most frequently located in the cerebellum and is characterized by slow growth and favorable prognosis when complete surgical resection is possible. Cerebellar pilocytic astrocytomas can cause obstructive hydrocephalus due to compression of the fourth ventricle or aqueduct of Sylvius, resulting in increased intracranial pressure and progressive neurological decline.

The coexistence of Fahr‐type intracranial calcification and a presumed cerebellar pilocytic astrocytoma is extremely rare, and the development of hydrocephalus in such a case adds further clinical complexity. Fahr‐type intracranial calcifications may complicate radiological interpretation, while the tumor‐induced hydrocephalus poses an urgent neurosurgical concern [1, 2, 6, 7, 8, 9]. Understanding the pathophysiological interplay between these two conditions is essential for accurate diagnosis, treatment planning, and prognostic evaluation.

## 2 Case Presentation

An 18‐year‐old female presented to the outpatient department with a 2–3‐year history of intermittent abdominal discomfort and recurrent vomiting, accompanied by mild to moderate headaches. Over the preceding 2–3 months, she experienced progressively worsening vision in both eyes. She developed complete loss of vision 5 days prior to presentation.

## 3 Clinical Examination

Higher mental functions: Normal.

Pupils: Bilaterally equal, mid‐dilated, reactive to light.

Visual function: Only fluctuating light perception in both eyes.

Motor examination: Normal power (5/5) in all four limbs.

Cerebellar signs: Positive finger–nose test on left side; impaired left rapid alternating movements.

There were no signs of limb ataxia or gait disturbance noted at the time of evaluation. Formal ophthalmologic assessment, including fundoscopy, dilated optic‐disc examination, and visual‐field testing, was not available, and no ophthalmology consultation was documented.

## 4 Neuroimaging Findings (Figures 1 and 2)

**FIGURE 1:**
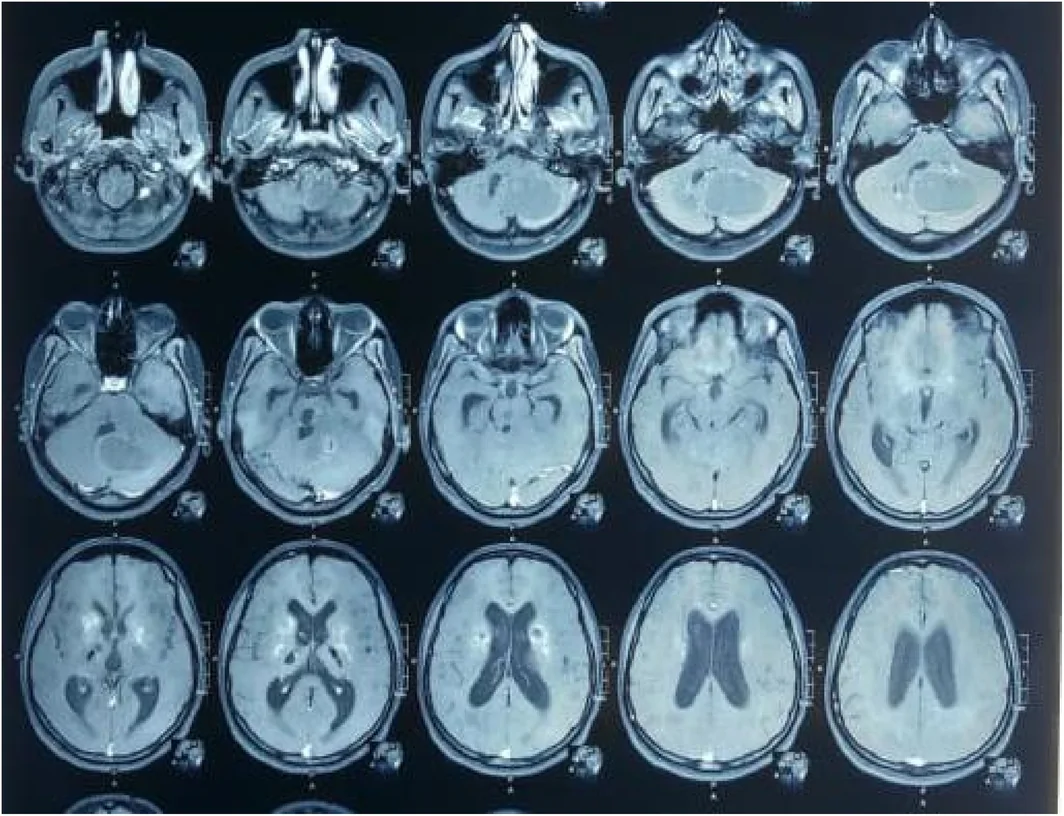
MRI Brain shows extensive bilateral basal ganglia and cerebellar calcification and cystic mass in left cerebellum compressing fourth ventricle producing obstructive hydrocephalus.

**FIGURE 2:**
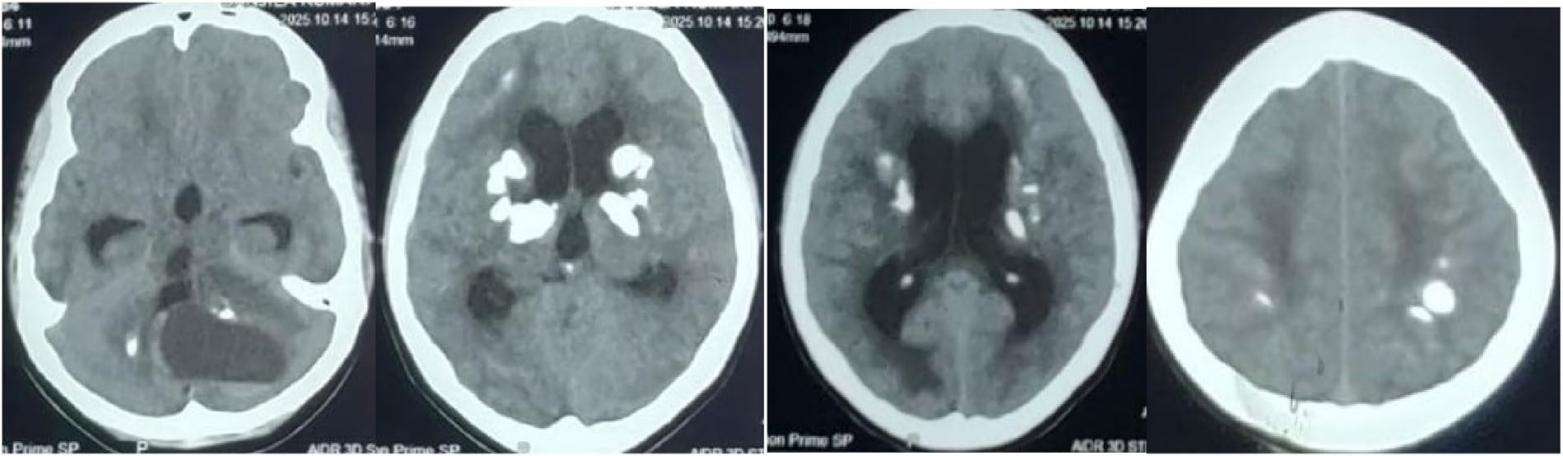
Pre‐op CT Scan of brain showing extensive bilateral basal ganglia, thalami, and cerebellar calcification and calcified cystic left cerebellar mass with hydrocephalus.

CT and MRI brain demonstrated: Extensive bilateral calcifications in the basal ganglia, thalami, cerebellar dentate nuclei → consistent with Fahr‐type intracranial calcification. A well‐defined cerebellar mass lesion with imaging features compatible with pilocytic astrocytoma.

Dilated ventricles and fourth ventricular obstruction consistent with obstructive hydrocephalus. Serum calcium, phosphate, and parathyroid hormone levels were not available, and genetic testing for the recognized causative genes was not performed, so the etiology of the intracranial calcification remained uncharacterized.

## 5 Management

**FIGURE 3:**
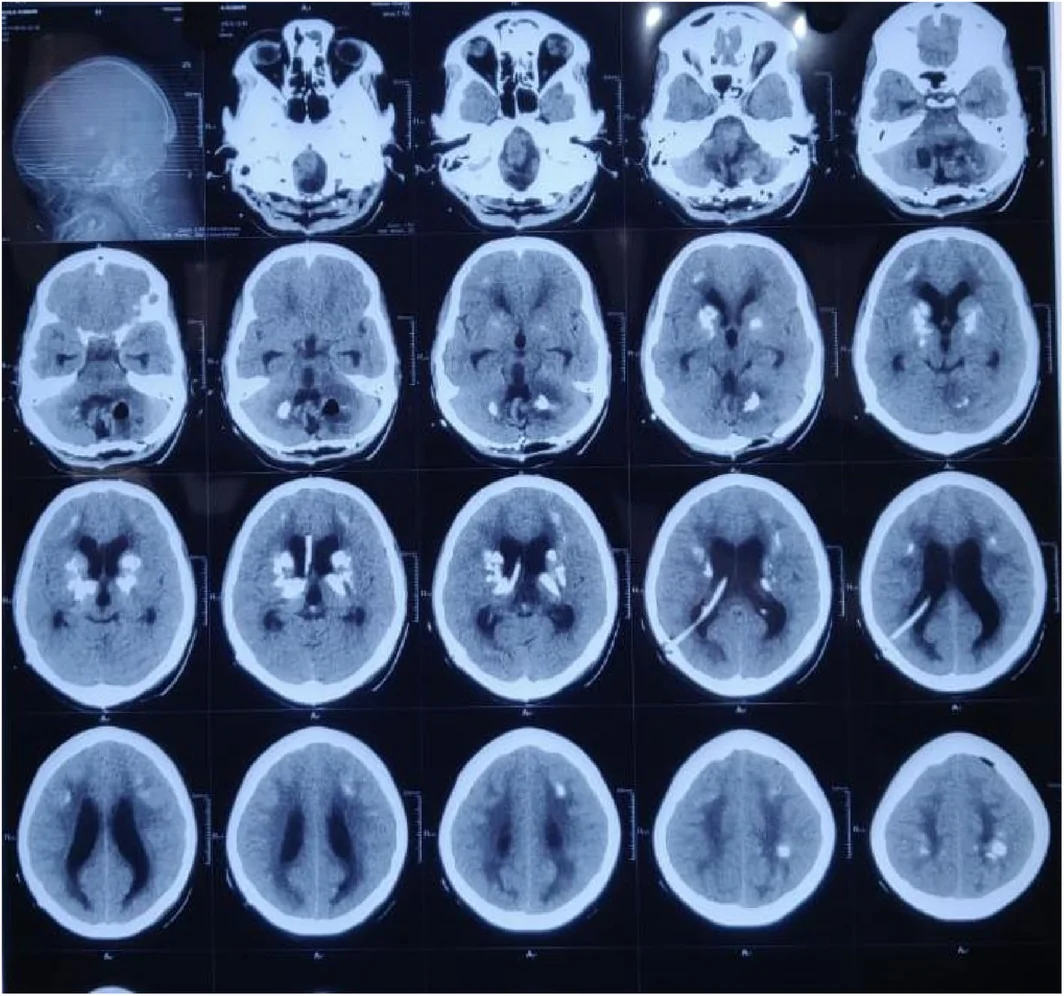
Post‐Op CT Scan of brain showing bilateral basal ganglia, thalami and cerebellar calcification with postoperative changes in sub‐occipital and cerebellar area with intra‐ventricular catheter in situ.

**FIGURE 4:**
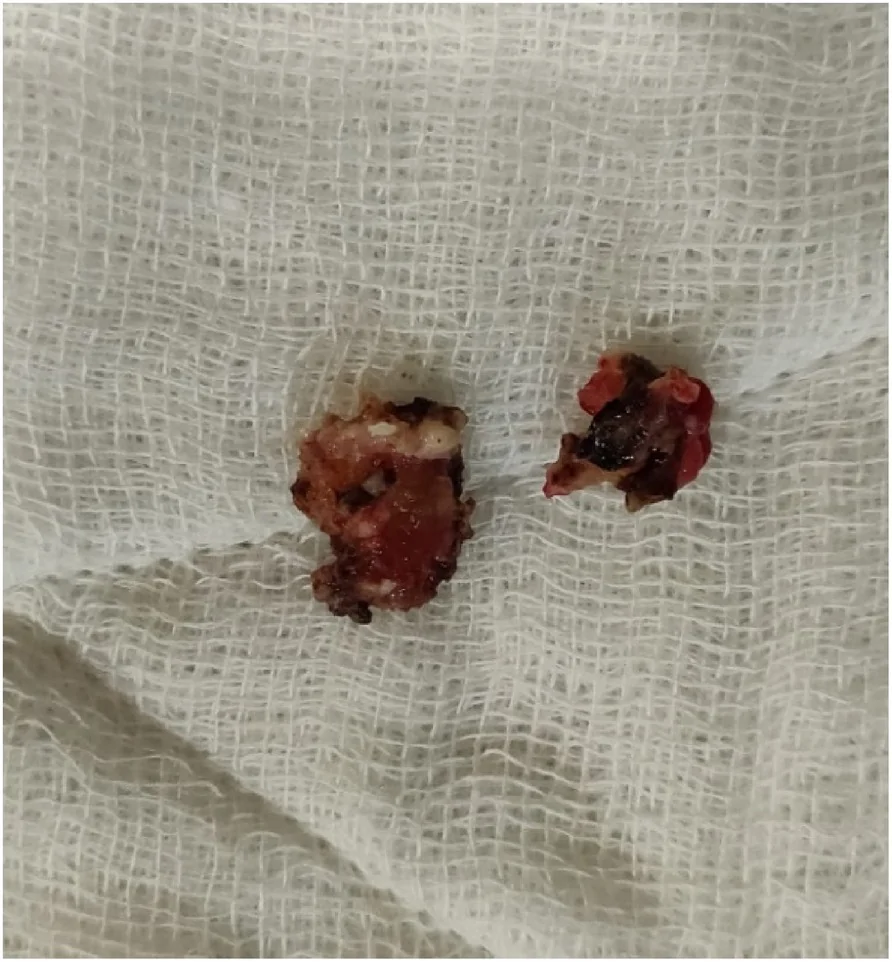
Excisional tumor tissue from left cerebellar mass.

**FIGURE 5:**
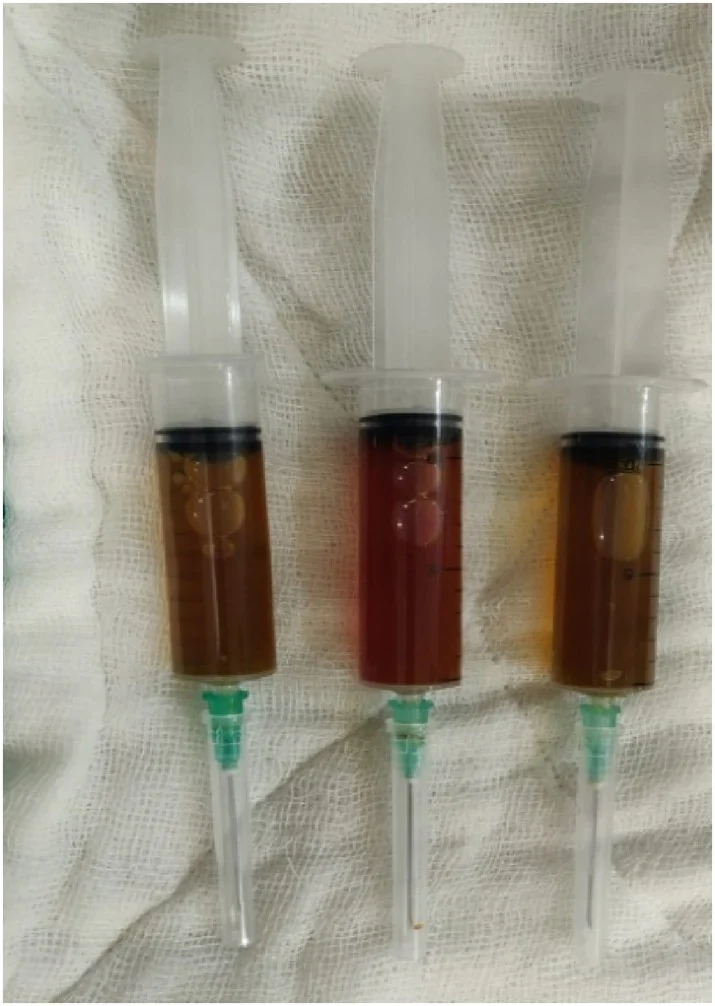
Cystic fluid from left cerebellar cystic mass.

The patient underwent suboccipital craniectomy with gross total excision of the left cerebellar mass on operative assessment, followed by ventriculoperitoneal (VP) shunt placement for hydrocephalus that persisted after tumor resection (Figures 3, 4, 5). The postoperative course was uneventful, and the patient showed gradual improvement in headaches and vomiting. Cerebrospinal fluid diversion for persistent hydrocephalus following posterior fossa tumor resection is a standard indication. The postoperative CT scan (Figure 3) demonstrated expected postoperative changes with the ventricular catheter in situ; the precise interval between resection and shunt placement and formal volumetric confirmation of resection extent were not recorded. Visual recovery, however, remained limited. A summary clinical timeline of the case is provided in Table 1.

**TABLE 1:** Clinical timeline.

Time point	Event
2–3 years before presentation	Intermittent abdominal discomfort, recurrent vomiting, and mild to moderate headaches
2–3 months before presentation	Progressively worsening vision in both eyes
5 days before presentation	Complete loss of vision
Presentation (admission)	Examination showed light perception only in both eyes and left cerebellar signs. CT and MRI showed extensive bilateral (Fahr‐type) calcification, a left cerebellar cystic mass, and obstructive hydrocephalus. Serum calcium, phosphate, and parathyroid hormone levels and genetic testing were not available; formal ophthalmologic assessment was not available
Surgery	Suboccipital craniectomy with gross total excision of the left cerebellar mass on operative assessment
Histopathology	Extensive hyalinization and regressive changes with largely acellular tissue; pilocytic astrocytoma considered a possible differential diagnosis. Immunohistochemistry and molecular testing were not available, so the diagnosis is presumptive
Postoperative	Ventriculoperitoneal shunt placement for hydrocephalus that persisted after tumor resection. Postoperative CT showed expected postoperative changes with the ventricular catheter in situ
Discharge	Gradual improvement in headache and vomiting; visual recovery remained limited
Follow‐up	Long‐term follow‐up is not yet available; continued surveillance for visual recovery, tumor recurrence, and progression of calcification is planned

Abbreviations: CT, computed tomography; MRI, magnetic resonance imaging.

**FIGURE 6:**
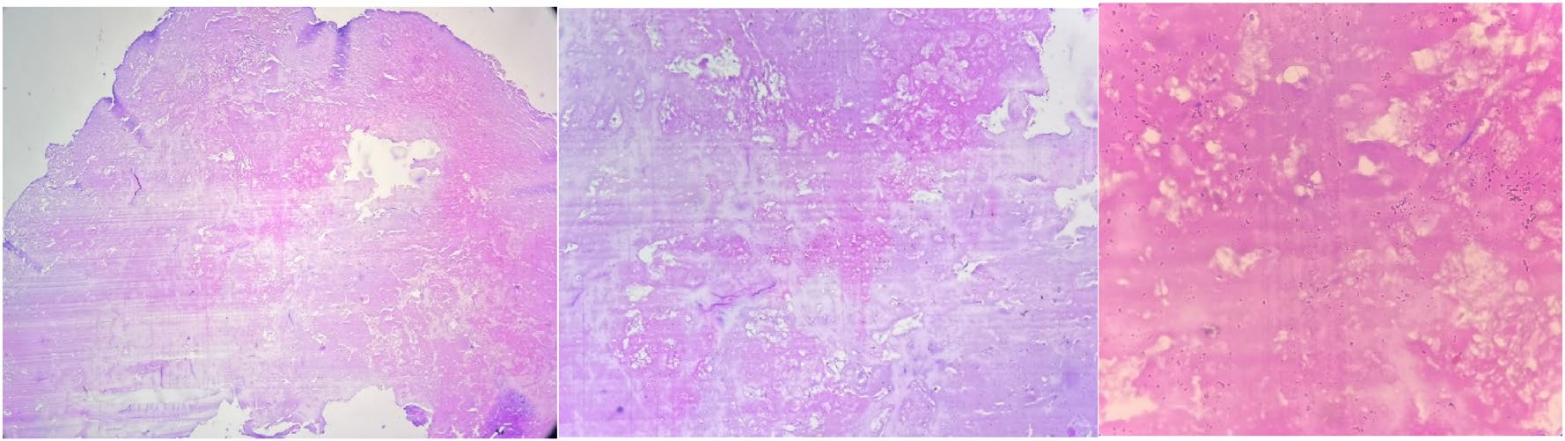
Histopathology report shows left cerebellar lesion. Comment: In the view of tissue with extensive hyalinization only without any visible cellular areas, regressive changes within. The pilocytic astrocytoma might be a possible differential diagnosis.

*Histopathology report* of the left cerebellar lesion showed tissue with extensive hyalinization and regressive changes, without any visible cellular areas. Pilocytic astrocytoma was considered a possible differential diagnosis (Figure 6). Immunohistochemistry and molecular testing were not available, so the diagnosis of pilocytic astrocytoma remained presumptive.

## 6 Discussion

This case represents an exceptionally rare coexistence of Fahr‐type intracranial calcification and a presumed cerebellar pilocytic astrocytoma, compounded by obstructive hydrocephalus. Calcifications associated with PFBC may obscure radiological interpretation of posterior fossa lesions, emphasizing the need for careful evaluation. Current evidence suggests no established causal relationship between PFBC and tumor development, making this combination likely coincidental [2, 7, 9, 10]. In previously reported cases, a consistent pattern has been observed, with relatively young patients and a predominantly cerebellar tumor location, where a cystic glioma arises adjacent to a calcified dentate nucleus [9]. Coexistence of PFBC with intracranial tumors, including gliomas and, more recently, meningioma, has generally been attributed to long‐standing reactive astroglial proliferation around calcified deposits rather than to a proven causal mechanism [7, 11, 12]. Importantly, regressive and degenerative features such as extensive vascular hyalinization, calcification, and paucicellular fibrillary areas are well recognized in pilocytic astrocytoma and can dominate the histological picture in long‐standing lesions [13]. In the present case, the biopsy showed hyalinized, largely acellular tissue, so the diagnosis remains presumptive; definitive confirmation would require characteristic bipolar piloid cells, Rosenthal fibers, and eosinophilic granular bodies, supported by GFAP immunohistochemistry and, where feasible, KIAA1549‐BRAF fusion testing, which is present in approximately 90% of cerebellar pilocytic astrocytomas [13]. Early diagnosis and timely surgical intervention are critical to preventing irreversible complications such as vision loss. Because posterior fossa tumors commonly precipitate obstructive hydrocephalus, resection of the mass is frequently the definitive treatment, with cerebrospinal fluid diversion reserved for persistent or symptomatic hydrocephalus, as in the present case [4]. The presenting feature in this patient, bilateral visual deterioration progressing to complete loss of vision, deserves particular emphasis. Posterior fossa masses rarely involve the visual pathway directly, and visual failure most likely reflects raised intracranial pressure transmitted to the optic nerve heads, where sustained papilledema can produce axoplasmic stasis, ganglion cell axonal loss, and ultimately optic atrophy [14, 15]. Because fundoscopic findings, the presence and grade of papilledema, and visual‐field data were not available in this patient, this mechanism is inferred rather than directly demonstrated. Papilledema usually resolves after tumor resection or cerebrospinal fluid diversion, but deficits that are severe or long‐standing at presentation may persist despite adequate treatment of the hydrocephalus, which accounts for the limited visual recovery observed in this patient [15]. Permanent visual loss is uncommon in posterior fossa tumors overall, reported in approximately 6 to 8% of children, and outcomes are generally most favorable for pilocytic astrocytoma. The profound and largely irreversible deficit seen here is therefore an atypical outcome, and it underscores the importance of early fundoscopic assessment and expedited cerebrospinal fluid diversion whenever visual symptoms accompany a posterior fossa mass [14]. A small number of comparable cases have been described, including those reviewed by Lin et al. and a coexistent meningioma reported in 2023; because our literature search was not systematic, we do not assign a precise total to previously reported cases [9, 12]. Published reports of Fahr disease concurrent with intracranial tumors (astrocytoma and meningioma) are summarized in Table 2 [7, 9, 12, 16].

**TABLE 2:** Individually reported cases of Fahr‐type intracranial calcification coexisting with intracranial tumors.

No.	Study (first author, journal, year)	Tumor type reported	Patient/key features (as reported)
1	Morimoto K, Mogami H, Ozaki K. Eur Neurol, 1984	Cystic astrocytoma	First described association (single patient). CT showed Fahr‐type calcification with an adjacent cystic astrocytoma.
2	Ang LC, et al. Surg Neurol, 1993	Astrocytoma/astrocytic proliferation (autopsy)	Low‐grade astrocytoma identified at autopsy with extensive bilateral calcification; astrocytic proliferation noted near calcified deposits
3	Lin Q, Piao Y, Ling F, Xu G. Exp Ther Med, 2015	Low‐grade astrocytoma	32‐year‐old woman with bilateral calcification and an adjacent low‐grade (cystic) astrocytoma; authors reviewed previously reported cases and noted a cerebellar/dentate adjacency pattern
4	Scalia G, et al. Radiol Case Rep, 2023	Meningioma	Coexistent meningioma in a patient with primary familial brain calcification; broadens the reported tumor types beyond astrocytoma
5	Present case, 2026	Presumed pilocytic astrocytoma	18‐year‐old woman with extensive bilateral (Fahr‐type) calcification and a left cerebellar cystic mass causing obstructive hydrocephalus. Histology showed hyalinized, largely acellular tissue, so the tumor diagnosis is presumptive

*Note:* Based on a non‐systematic literature search; the list is illustrative rather than exhaustive, and no precise total of previously reported cases is implied.

Abbreviations: CT, computed tomography; PFBC, primary familial brain calcification.

## 7 Limitations

This report has several limitations. First, serum calcium, phosphate, and parathyroid hormone levels were not available, so calcification secondary to a disorder of calcium metabolism could not be excluded biochemically and the case cannot be classified with certainty as primary familial brain calcification rather than Fahr syndrome. Second, no family history was documented and genetic testing for the recognized causative genes was not undertaken, so the designation of PFBC remains radiological rather than molecularly confirmed. Third, the resected specimen showed only hyalinized, largely acellular tissue, and neither immunohistochemistry nor molecular testing was available, so the diagnosis of pilocytic astrocytoma remains presumptive. Fourth, long‐term follow‐up is not yet available, and continued surveillance is required to assess visual recovery, tumor recurrence, and progression of calcification. In addition, formal ophthalmologic assessment, including fundoscopy, grading of papilledema, and visual‐field testing, was not available, so the mechanism of visual loss is inferred rather than directly documented. Finally, observations drawn from a single case cannot establish a causal relationship between the two conditions.

## 8 Conclusion

The coexistence of Fahr‐type intracranial calcification and a presumed cerebellar pilocytic astrocytoma presenting with obstructive hydrocephalus is rare. Because the etiology of the calcification was not characterized and the tumor diagnosis remained presumptive, this report describes an association rather than a proven causal link, and it underscores the importance of matching diagnostic language to the available evidence and of considering more than one pathology when symptoms are progressive and disproportionate to a single diagnosis. Increased reporting of comparable cases may clarify whether any relationship exists between intracranial calcification disorders and intracranial tumors.

## Author Contributions

**Bikramaditya Prasad Sah:** conceptualization, investigation, writing – original draft, methodology, data curation. **Kashif Qureshi:** visualization. **Murtaja Satea:** visualization. **Khaled Fares AlAli:** visualization. **Maren Ruka:** visualization. **Muhammad Mohsin Khan:** visualization. **Tural Rajabov:** visualization. **Bipin Chaurasia:** writing – review and editing, visualization, validation, supervision.

## Funding

The authors have nothing to report.

## Disclosure

*Guideline Compliance*: This case report was prepared in accordance with the CARE guidelines for case reports.

## Ethics Statement

Ethical approval was not required for this case report in accordance with the institutional policy of Madan Bhandari Academy of Health Sciences, Hetauda Hospital.

## Consent

Written informed consent was obtained from the patient prior to inclusion. Written informed consent was obtained from the patient for publication of this case report and any accompanying images.

## Conflicts of Interest

The authors declare no conflicts of interest.

## Data Availability

The data that support the findings of this study are available from the corresponding author upon reasonable request.
